# Defensin alpha 6 (*DEFA 6*) overexpression threshold of over 60 fold can distinguish between adenoma and fully blown colon carcinoma in individual patients

**DOI:** 10.1186/1471-2407-10-588

**Published:** 2010-10-27

**Authors:** Mariya Y Radeva, Franziska Jahns, Anne Wilhelm, Michael Glei, Utz Settmacher, Karl Otto Greulich, Henning Mothes

**Affiliations:** 1 Leibniz Institute for Age Research - Fritz Lipmann Institute, Beutenbergstr. 11, 07745 Jena, Germany; 2 Friedrich Schiller University Jena, Department of Nutritional Toxicology, Dornburger Str. 24, 07743 Jena, Germany; 3 University Hospital Jena, Department of General, Visceral and Vascular Surgery, Erlanger Allee 101, 07747 Jena, Germany

## Abstract

**Background:**

It is known that alpha-defensin expression is enhanced in colon cancer. However, the expression of human alpha defensin 6 (DEFA 6) in earlier stages, such as adenoma, has so far not yet been studied in a patient resolved manner.

**Methods:**

By using quantitative Real Time-PCR, the gene expression pattern of *DEFA 1-3 *and *DEFA 6 *was analyzed in tissue of different stages of carcinogenesis, derived from colorectal cancer patients. In addition to paired normal and tumor tissue, matched normal near tumor and adenoma tissue samples were examined.

**Results:**

The median gene expression of human defensin alpha 6 (*DEFA 6*) has been found to be moderately increased (~ 5 fold) in tumor samples derived from individuals with colorectal cancer (CRC) when compared to their normal counterparts. However, when the data were analyzed in a patient-wise manner, a large expression variation among individual patients is found, making the use of *DEFA 6 *for individual diagnosis of fully blown colon carcinoma difficult. Surprisingly, in adenoma the gene expression analysis revealed a 100 fold increased median expression of *DEFA 6 *relative to normal colon tissue. 13/18 samples had an individual overexpression of more than 60 fold in adenoma but only 3/17 in carcinoma. In each of the individual patients, at least either the adenoma or the carcinoma showed strong *DEFA 6 *overexpression.

**Conclusions:**

We suggest that the expression of *DEFA 6 *preferably can be used as a potential diagnostic marker for adenoma and not as a marker for fully blown carcinoma. This is supported by the fact that *DEFA 6 *is a downstream target of the Wnt pathway, which is mutational active during the earliest stage of cancer development.

## Background

Human neutrophilic (DEFA 1-3) and enteric (DEFA 6) alpha defensins are cationic short peptides of 29 to 42 amino acids in length with known functional activities in innate antimicrobial immunity [1-3]. While DEFA 1-3 are major components of the dense azurophilic granules of neutrophils, DEFA 6 is primarily expressed in the lysozyme-rich granules of the Paneth cells of the small intestine [4], but also found in intermediate cells [5]. As involved in the host defense of the gut, the expression of alpha defensins is usually found to be elevated in chronically inflamed colon, but not in the respective healthy tissues [5,6]. Furthermore, several studies already reported the elevated alpha defensin expression in various tumour types, suggesting their potential to be used as tumour markers [6-9]. Their expression was shown to be enhanced in fully blown colon cancer tissue, though an evaluation of individual patient's data has not yet been reported [10-12]. More interestingly, in contrast to DEFA 1-3, which has been shown to be an unspecific colon cancer marker, DEFA 6 is more tissue specific due to its high expression in colon cancer as compared to other tumors [11].

This study attempts to test whether DEFA 6 can be used as marker of the adenoma stage of colon cancer and not solely, as already described in literature, as a marker of fully blown colorectal cancer. The emphasis is on evaluating the data on an individual patient's basis before pooling the data, since a high median overexpression, at large variation of the individual patient's data, would not be really suited for a diagnosis.

## Methods

### Tissue sample preparation for gene expression analysis

Tissue samples were prepared as described in Radeva et al [13]. In short, the normal colon samples from each patient with colon cancer were taken at a distance of 20 to 50 cm from the tumor site. Confirmation of the tumor stage of the patients was provided by pathological examination after the surgery (Table 1). In addition to the adenoma, macroscopically normal tissues, removed at distance 1 to 5 mm apart from the tumor were collected. All of the obtained adenoma samples were benign. Only hyperplastic polyps and villous or tubular adenomas were taken, but not flat serratous adenomas since these might have different pathology.

**Table 1:** Clinicopathological characteristics of 17 patients.

Patient and tumor characteristics	Number of cases
Age	Mean age 68.7 ± 9.3
Gender	13 males, 4 females
Type of analyzed tissue *	
Normal	17
Normal near Tumor	9
Adenoma	18**
Tumor	17*** (1 with G1, 6 with G2; 8 with G3; 1 with G4)

* Four different types of tissues were examined for expression of *DEFA 1-3*, *DEFA 6 *and *β- actin *genes.

** One donor has two types of adenoma

*** For 1 out of 17 analyzed donors the clinical description about the grade of the tumor was not available. In addition, 1 out of the total 17 donors investigated does not possess tumor tissue.

### Ethics

The research was carried out in compliance with the Helsinki Declaration. Furthermore, the study was institutionally approved by the ethics committee of University Hospital of Jena, Germany (Reference number 1601-08/05). Prior to tissue collection, verbal consent was obtained from all analysed patients.

### Total RNA extraction from tissue samples. cDNA preparation

Total cellular RNA preparation was performed as described in Radeva et al [13]. In short, RNA was isolated from epithelium colon stripes using RNeasy Mini Kit (Qiagen, Hilden, Germany). Integrity of the isolated total RNA was checked by using an Agilent 2100 bioanalyzer (Agilent Technologies, Palo Alto, CA, USA). cDNA synthesis was performed as described earlier [13]. However, due to the variability of the quantity of the extracted total RNA, the amount used as an initial material for the performance of the cDNA synthesis varied (ranged from 10 ng to 300 ng total RNA).

### Design of primers

All primer pairs were designed using freely available Primer3 software, version 0.4.0 http://frodo.wi.mit.edu/. To assess the primer specificity, basic local aligment search tools (BLAST, http://blast.ncbi.nlm.nih.gov/Blast.cgi) were applied. The amplicon size varied within the range from 160 bp to 222 bp. Sequences of the used primer pairs are shown in Table 2. Besides the lack of *DEFA 2 *gene, the sequence of the genes encoding *DEFA 1 *and *DEFA 3 *differ by only 2 nucleotide substitutions [14], therefore one primer pair was used for detection of all neutrophilic defensins. For further verification of the primer specificity, the products amplified by defensin's primer pairs were sequenced.

**Table 2:** Sequence of oligonucleotide primers used for PCR amplification and product size predicted for sample cDNA.

Gene name	Gene annotation	RefSeq ID	Sequence	Amplicon size
DEFA 1-3*	Defensin, alpha 1 to 3	NM_005218.3	CCTGCCTAGCTAGAGGATCTGTG	222 bp
		NM_005217.2	TGTTTTTCCTTGAGCCTGGA	
				
DEFA 6*	Defensin, alpha 6, Paneth cell-specific	NM_001926.2	CTCAAGTCTTAGAGCTTTGGGCT	198 bp
			GGACACACGACAGTTTCCTTC	
β-actin*	Beta actin	NM_001101	AGAGCCTCGCCTTTGCCGAT	160 bp
			CCCACGATGGAGGGGAAGAC	

* All primers are listed as 5' to 3'.

### Verification of the primer's specificity

To verify the primer specificity and to ensure that the designed primer pairs were not amplifying additional products in the presence of genomic DNA, *in-silico *PCR, http://genome.ucsc.edu/cgi-bin/hgPcr?command=start was implemented. In addition to this computational procedure, experimental approach such as standard polymerase chain reaction (PCR) was carried out. For generation of the amplicon, reflecting the relative abundance of certain target gene, the gene specific primers were tested in a presence of various templates, such as genomic DNA (gDNA) and complementary DNA (cDNA). Non-template control reaction (NTC) that contains all essential components of the amplification reaction except the template enables detection of contaminations.

**Figure 1:**
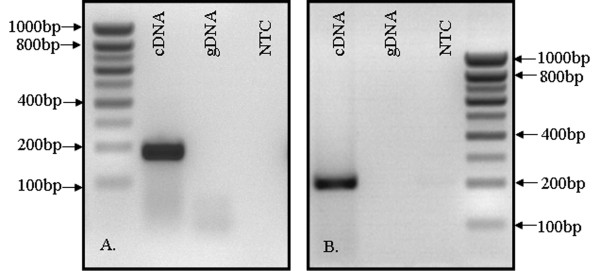
**Verification of primer specificity**. (A) Defensin alpha 6 (*DEFA 6*) and (B) Defensin alpha 1-3 (*DEFA 1-3*). The expected amplicon size is 198 bp and 222 bp, respectively. In order to verify the lack of contaminations, non-template control reaction (NTC) was run in parallel.

Amplification was performed using Go Tag DNA Polymerase (Promega) with the following thermal conditions: 95°C for 2 min followed by 40 cycles of 95°C for 30 sec, 60°C for 30 sec and 72°C for 30 sec and as a last step, final extension at 72°C for 10 min was run. On Figure 1, representative examples are shown. If the primers are specific, amplicon with correct size should be observed only in case where cDNA was used as a DNA template.

### PCR reaction efficiency

Validation of the PCR reaction efficiency and the performance of the quantitative RT-PCR assay was done as previously described [13]. Two microliters cDNA prepared from different starting concentration of total RNA was used as a template. Prior further evaluation, the data was additionally normalized to definite starting concentration in order to remove the errors introduces due to uneven sample quantities. The normalization algorithm is available from the authors upon request.

### Statistical analysis

All samples were amplified in duplicate or triplicate and the means were obtained for further calculations. In all samples analyzed, the mRNA of each target gene was normalized to that of the *β-actin *mRNA. The relative RNA expression was calculated using the following formula:

$$2^{\left(\text{Ct reference-Ct gene of interest}\right)}$$

where Ct corresponds to the number of cycles needed to generate a fluorescent signal above a predefined threshold. The averaged Ct value for the reference gene was subtracted from the averaged Ct value of the selected gene of interest.

The data was exported to GraphPad Prism Software, version 4 (Graph Pad, San Diego, USA). Comparisons between two groups were made using Wilcoxon matched pairs test. Overall differences between multiple groups were tested using the nonparametric Kruskal-Wallis test.

## Results

### Gene expression profiling of human subjects

In the present work, the expression of *DEFA 6 *gene was tested and compared with the respective expression of *DEFA 1-3 *genes. For this purpose, tissue samples of 17 CRC patients were studied. Since the gene expression analysis of fully blown carcinoma does not account for the expression alterations that are critical for the initiation and the development of cancer, in addition to the classical combination of paired normal and tumor tissue, also matched normal near tumor and adenoma tissue samples were examined by qRT-PCR.

### Low abundance of *DEFA 1-3 *expression in colon tissues

**Figure 2:**
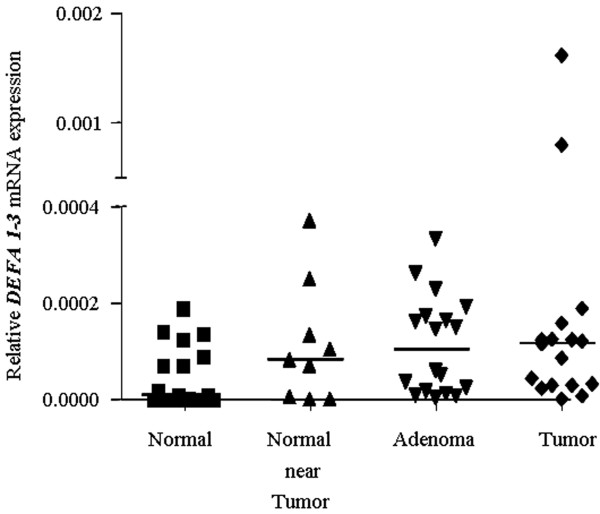
**Gene expression of *DEFA 1-3***. The *DEFA 1-3 *expression was normalized to *β-actin*, in normal, normal near tumor, adenoma and the respective tumor tissue samples. For better visualization the scale was segmented with suppression of the highest values. The horizontal lines represent the median over all investigated patients. Differences between multiple groups were tested using Kruskal-Wallis test. Significant up-regulation was observed in adenoma tissue over the related normal colon tissue (* p < 0.05). The rest of the comparisons did not show significance according to the test (p > 0.05).

The expression of *DEFA 1-3 *genes was investigated in normal and the related normal near tumor, benign adenoma and tumor tissue samples derived from 17 patients in total. Resume of the performed quantitative Real Time-PCR is presented in Figure 2.

Transcription levels of *DEFA 1-3 *mRNA, in all colon tissues investigated were relatively low, when compared to the expression of *DEFA 6 *(see below). Although low abundant, *DEFA 1-3 *mRNA was significantly up-regulated in adenoma and tumor tissues compared to the normal samples with ~ 4- and 6.4-fold, respectively (Wilcoxon test, p = 0.0007 (adenoma vs. normal), p = 0.02 (tumor vs. normal)). Moreover, the expression in normal near tumor was also increased by factor of 2.8 over normal tissue (Wilcoxon test, p = 0.04). The expression alterations among normal near tumor, adenoma and tumor colon tissues were relatively small, in the range of 1.33 to 1.4 fold, and according to the performed Wilcoxon matched pairs test all of these differences were not statistically significant (p > 0.5).

In summary, the presented results verified the general observation of *DEFA 1-3 *elevation in colon tumors, which consist of a mix of different cell populations such as epithelial cells, a small percentage of fibroblasts and leukocytes. Therefore, it is still unclear whether the invasion of the neutrophils or the defensin production by the cancer cells themselves is the reason for the altered DEFA 1-3 expression in colon cancer tissues samples [6,8,15,16].

### A burst of *DEFA 6 *in benign adenoma

**Figure 3:**
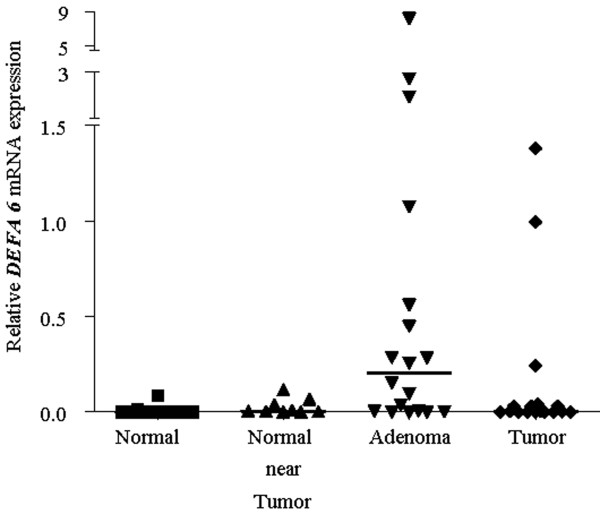
**Relative gene expression of *DEFA 6 *as already described for Figure 2**. According to the performed Kruskal-Wallis test, the median expression in adenoma as compared to normal tissue was significantly elevated (p< 0.001).

As for *DEFA 1-3*, the expression of *DEFA 6 *gene was investigated across the adenoma-carcinoma axis. The outcome of the analysis is summarized in Figure 3.

The increase in *DEFA 6 *expression when compared to the rest of the tested groups is immediately evident. The horizontal lines represent the median expression of the population analyzed indicating significant increase with a 100 fold in adenoma over normal tissue (Wilcoxon test, p = 0.0004). Still large was the increase of *DEFA 6 *in adenoma over the normal near tumor tissue (30.6 fold), and this expression's alteration, according to the applied Wilcoxon test, was significant (p = 0.02). When the median expression in tumor was compared with that in normal tissues, moderate increase with almost 5 fold was detected in fully blown tumors than in the non-malignant, normal samples, and those differences, based on the performed statistical test, were significant (p = 0.012).

**Figure 4:**
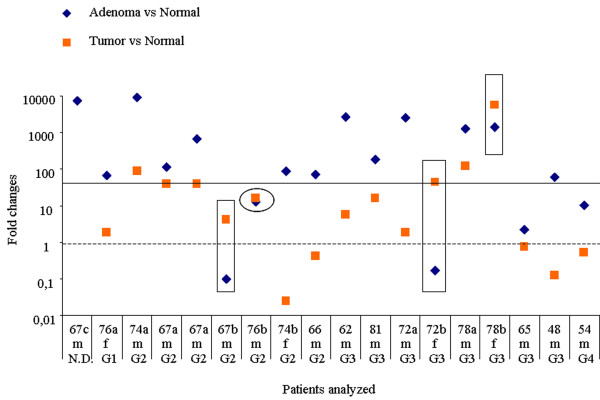
Up or down - regulation of *DEFA 6 *expression in 17 individuals*. *One of the donors had two adenomas, therefore the figure show 18 samples. The patients are characterized for their age, gender and grade of cancer. N.D. means that the stage was not determined. The scale of the y axis is logarithmic. None of the patients has simultaneously down-regulated *DEFA 6 *in both, adenoma as well as fully blown cancer. The dotted horizontal line at 1 indicates that *DEFA 6 *expression would be un-effected. For the first patient on the left, no comparison of adenoma with carcinoma was possible, since the donor had developed only adenoma. From the 17 other samples, 13 revealed a higher expression in adenoma than in carcinoma, 1 case (encircled) showed essentially no change and 3 objects (in rectangular boxes) had the highest expression in carcinoma.

A 100 fold average increase in one single stage of colon cancer development is a comparably strong genetic marker. However, the full potential of these data becomes only evident when it is presented in a patient specific manner. Thus, Figure 4 summarizes the computed *DEFA 6 *expression ratio between adenoma and normal (rhombs) as well as between tumor and normal (squares) colon tissue, obtained from each single individual analyzed. The patient's characteristic such as age, gender and grade are denoted below the figure. No consistent dependence of *DEFA 6 *expression ratios as a function of tumor grade, age or gender has been detected. The ratios in adenoma as well as in carcinoma as compared to normal tissue vary more than 1000 fold indicating the individuality of each patient's cancer. However, *DEFA 6 *is overexpressed in all cases either in adenoma or carcinoma - which indeed means that all those tissue samples can be characterized as no longer normal. In addition, in 13/18 cases (72%) an overexpression of more than 60 fold indicates an adenoma (see Figure 4, the black horizontal line is empirical and separates all samples with overexpression of 60 or more fold from the rest of the samples). Most of the tumor tissues showed overexpression with less than 60 fold. In addition, we did not find a correlation between the *DEFA 6 *expression in adenoma and carcinoma which might be systematically related to the grade of the tumor.

## Discussion

Enhanced alpha-defensin gene expression can be detected in samples such as stool [16], serum, plasma [6,10] and colon tissue [11,12,15] derived from colorectal cancer patients. In this sense, our results on the *DEFA 6 *gene in colon cancer tissues is in agreement with Nam et al.. There by ELISA, DEFA 6 was also found to be enhanced on the protein expression level in serum from colorectal cancer patients indicating a good correlation between DEFA 6 mRNA and protein expression. DEFA 6 showed even a higher diagnostic sensitivity and specificity than CEA, the most widely used marker for colon cancer diagnosis [11]. Thus, the observed stable correlation between DEFA 6 mRNA and protein expression indicates that no dramatic posttranscriptional regulation occurs. It would also be interesting to address the question what are the serum DEFA 6 expression levels of patients with adenoma only. However, such patients are difficult to be obtained due to the fact that in most of the cases the patient is diagnosed too late, when the carcinoma has been already fully developed.

In adenoma, we detected extremely high *DEFA 6 *expression in almost all individual patients. This makes *DEFA 6 *a suitable target for diagnosis of individuals. In 12 out of 17 samples the effect of *DEFA 6 *overexpression in adenoma was reverted in the fully blown tumor. Thus, *DEFA 6 *expression in tumor was distinctly lower compared to adenoma, but still remained higher than in normal tissue.

To our best knowledge this is the first study demonstrating a gene expression explosion of *DEFA 6 *in premalignant adenoma obtained from patients with colorectal cancer. This striking result suggest that the high *DEFA 6 *overexpression is the hallmark of adenoma, since the expression threshold of 60 fold (see Figure 4) discriminates adenoma from carcinoma in a sharper way than many other disease markers for individual patients and may thus be envisioned as a simple auxiliary diagnostic tool for the clinical histologist.

Neoplastic tissue is well known to be heterogeneous. Thus, a large proportion of adenoma and carcinoma cells showed different expression levels of various targets under investigation. As an example, Ki-67 and Myc, known markers for proliferation, were found to be overexpressed in areas of increased cell proliferation [17,18]. On other hand, Andreu et. al reported that the *DEFA 6 *mRNA expression level was elevated in tumors, where Myc and Cyclin D were accumulated as well [19]. This is not surprising due to the fact that the three genes are known to be down stream targets of the same pathway, namely Wnt pathway. Therefore, if within the tumor the mRNA expression of *DEFA 6 *is in stable positive correlation with the mRNA expression of well known proliferation markers, such as Myc, strong DEFA 6 expression can be seen in areas of the tumor associated with a higher proliferation.

## Conclusions

To our knowledge this is the first study demonstrating a strong burst of the *DEFA 6 *gene in human adenoma tissue samples. This observation immediately indicates the potential of DEFA 6 to be used as a marker for early premalignant stages of colorectal cancer and not solely as a marker for colon cancer detection.

## Competing interests

The authors declare that they have no competing interests.

## Authors' contributions

MR designed the experiments, carried out the main part of the studies with patient material as well as MR performed the statistical analysis and wrote the manuscript. FJ and AW participated in the performance of the gene expression analysis of patient material. HM and US performed the surgery and provided the colon tissue material. KOG and MG initiated the study, participated in its supervision, design and coordination as well. All authors have read and approved the final manuscript.

## Pre-publication history

The pre-publication history for this paper can be accessed here:

http://www.biomedcentral.com/1471-2407/10/588/prepub
